# Function and quality of life following stroke rehabilitation: have our stroke patients gained optimum recovery?

**DOI:** 10.1186/1471-2458-12-S2-A7

**Published:** 2012-11-27

**Authors:** Nor Azlin Mohd Nordin, Noor Azah Aziz, Saperi Sulong, Syed Mohamed Aljunid

**Affiliations:** 1 Faculty of Health Sciences, Universiti Kebangsaan Malaysia, Jalan Raja Muda Abdul Aziz, 50300 Kuala Lumpur, Malaysia; 2 United Nations University-International Institute for Global Health, Universiti Kebangsaan Malaysia Medical Centre, Jalan Yaacob Latiff, 56000 Kuala Lumpur, Malaysia; 3 Department of Family Medicine, Universiti Kebangsaan Malaysia Medical Centre, Jalan Yaacob Latiff, 56000 Kuala Lumpur, Malaysia; 4 Department of Health Information, Universiti Kebangsaan Malaysia Medical Centre, Jalan Yaacob Latiff, 56000 Kuala Lumpur, Malaysia

## Background

There is limited research data on post-rehabilitation function and quality of life despite the increasing role of rehabilitation in the care of stroke patients in Malaysia. Outcome data is important in evaluating the effectiveness of stroke rehabilitation services in the country.

## Aims

The aim of this study was to assess function and quality of life in stroke patients following intensive rehabilitation at a tertiary hospital.

## Materials and methods

This was a cross-sectional study of 91 stroke patients; mean age 58.9±10.6 years, 79% male, median stroke duration 13 months who have completed intensive individual rehabilitation at the Universiti Kebangsaan Malaysia Medical Centre in the years 2010 and 2011. Rehabilitation outcome was measured with the use of standardised tools; Rivermead Mobility Scale (RMI), Berg’s Balance Scale (BBS), Sit to Stand Test (STS) for lower limb strength and Timed 10 metre walk test for walking speed. Post-rehabilitation disability level and quality of life were also assessed on a Modified Rankin Scale (mRS) and Euro-Qol 5 Dimensions-Visual analogue Scale (EQ5D-VAS), respectively. All data were analysed descriptively using SPSS version 18.

## Results

The median duration of rehabilitation was 10.5 months (range 5-25) in the study patients and post-rehabilitation mean mRS was 2.3±0.7. The median RMI was 13 (range 6-15), median BBS 51 (range 20-56) and median STS 15.5 secs (range 7.9-83.9 secs). The EQ5D-VAS mean score was 71.5±17 and mean walking speed at the completion of intensive rehabilitation was 49.4±28.3 m/min; less 22 m/min when compared with the optimum walking speed required for safe road crossing.

## Conclusion

Although our stroke patients gained satisfactory levels of mobility, balance and strength following intensive rehabilitation, they have not achieved optimum speed of walking to enable effective community ambulation. Prolongation of rehabilitation programme may assist in further functional and quality of life gain among the post-stroke patients.

